# Comparison of culture media for ex vivo cultivation of limbal epithelial progenitor cells

**Published:** 2013-01-17

**Authors:** Renata Ruoco Loureiro, Priscila Cardoso Cristovam, Caio Marques Martins, Joyce Luciana Covre, Juliana Aparecida Sobrinho, José Reinaldo da Silva Ricardo, Rossen Myhailov Hazarbassanov, Ana Luisa Höfling-Lima, Rubens Belfort, Mauro Nishi, José Álvaro Pereira Gomes

**Affiliations:** 1 Ocular Surface Advanced Center (CASO), Federal University of São Paulo, São Paulo, Brazil; 2 Cornea and External Disease Service, Department of Ophthalmology, Federal University of São Paulo, São Paulo, Brazil

## Abstract

**Purpose:**

To compare the effectiveness of three culture media for growth, proliferation, differentiation, and viability of ex vivo cultured limbal epithelial progenitor cells.

**Methods:**

Limbal epithelial progenitor cell cultures were established from ten human corneal rims and grew on plastic wells in three culture media: supplemental hormonal epithelial medium (SHEM), keratinocyte serum-free medium (KSFM), and Epilife. The performance of culturing limbal epithelial progenitor cells in each medium was evaluated according to the following parameters: growth area of epithelial migration; immunocytochemistry for adenosine 5′-triphosphate-binding cassette member 2 (ABCG2), p63, Ki67, cytokeratin 3 (CK3), and vimentin (VMT) and real-time reverse transcription polymerase chain reaction (RT–PCR) for CK3, ABCG2, and p63, and cell viability using Hoechst staining.

**Results:**

Limbal epithelial progenitor cells cultivated in SHEM showed a tendency to faster migration, compared to KSFM and Epilife. Immunocytochemical analysis showed that proliferated cells in the SHEM had lower expression for markers related to progenitor epithelial cells (ABCG2) and putative progenitor cells (p63), and a higher percentage of positive cells for differentiated epithelium (CK3) when compared to KSFM and Epilife. In PCR analysis, ABCG2 expression was statistically higher for Epilife compared to SHEM. Expression of p63 was statistically higher for Epilife compared to SHEM and KSFM. However, CK3 expression was statistically lower for KSFM compared to SHEM.

**Conclusions:**

Based on our findings, we concluded that cells cultured in KSFM and Epilife media presented a higher percentage of limbal epithelial progenitor cells, compared to SHEM.

## Introduction

Previous studies have shown the maintenance of the corneal epithelial cell mass as determined by a distinct population of unipotent stem cells (SCs), which is probably located in the basal layer of the corneoscleral limbal epithelium [1,2]. These cells simultaneously retain their capacity for self-renewal and maintain a constant cell number by giving rise to fast-dividing progenitor cells, termed transit-amplifying cells (TAC), which proliferate and differentiate into post-mitotic corneal epithelia [3].

Various pathologic conditions that affect the ocular surface can partially or completely destroy the limbal epithelial SC repository giving rise to what is called limbal SC deficiency [4]. Total limbal SC deficiency (TLSCD) is characterized by 3,600 conjunctival epithelial ingrowth, vascularization, chronic inflammation, recurrent erosions, and destruction of the basement membrane, leading to severe functional impairment [4–8]. A renewal of the limbal epithelial progenitor cells population is required for regenerating the transparent corneal surface and restoring visual function in these eyes [9–11].

Limbal epithelial transplantation has allowed us to treat TLSCD with an acceptable anatomic and functional outcome [4]. However, autologous limbal epithelial transplantation is limited by the availability of limbal tissue from the same patient, and allogeneic transplantation requires systemic immune suppression to improve graft survival [1,4,8,11].

Alternatively, ex vivo cultivation and transplantation of limbal epithelial cells have been reported in animal models and in patients with TLSCD with variable results [1]. Questions related to the percentage of progenitor cells transplanted and their adhesion, survival, and mechanism of action have been raised [9,12,13]. This variation in the clinical outcome observed by investigators may be explained by differences in the culture techniques [14]. These differences include the use of explant or single-cell suspension systems, the presence or absence of a 3T3 feeder layer, the use of different carriers including fibrin and amniotic membrane, and the use of airlifting to promote epithelial differentiation and stratification [1,6,14–19].

In most cases, the current methods used to establish the cultures do not favor preserving stemness, but promote proliferation and terminal differentiation of TAC [3,16,19]. The long-term restoration of the damaged ocular surface, however, may require preserving limbal epithelial progenitor cells during the culture process and post-grafting [5,6,8,10,13,16]. With this background in mind, we aimed to explore the effectiveness of three culture media (SHEM, KSFM, and Epilife) for the growth, proliferation, differentiation, and viability of ex vivo cultured limbal epithelial progenitor cells.

## Methods

### Explant culture

Limbal tissue was obtained from ten human corneal rims of the remaining trephination of in penetrating keratoplasty at the Operating Room at the Department of Ophthalmology, Federal University of São Paulo (UNIFESP). Corneal rims were transported in Optisol GS (Chiron Ophthalmics, Irvine, CA) to the cell biology laboratory (Ocular Surface Advance Center, CASO). The research protocol was previously approved by the Institutional Ethics Committee for Research of the Federal University of São Paulo (UNIFESP), São Paulo, Brazil, in accordance with the tenets of the Declaration of Helsinki for experiments involving human tissue.

Each donor corneoscleral rim was divided into six equal pieces, in a laminar flow chamber, under aseptic conditions [12,20] and using a dissecting microscope. The endothelial and posterior stromal layers were carefully peeled off, and each explant was placed, with the epithelial surface facing upward, on a six-well 35 mm plate, one piece per well (TPP, Zurich, Switzerland). The explants were left on the covered plate for approximately 15 min under laminar flow and then cultured with one of the three studied culture media. Afterward, 1 ml of each culture medium to be tested was carefully placed on the explants. The cultures were maintained in a 37 °C humidified incubator with 5% CO_2_. The medium was changed three times a week for 4 weeks, and the explants were left in the culture plates during the incubation period.

### Culture media

The following culture media were used to cultivate limbal epithelial cells: A) The first was supplemental hormonal epithelial medium (SHEM), a combination of Dulbecco’s Modified Eagle’s Medium/Ham’s F-12 nutrient mixture (DMEM/F12; Invitrogen, Gibco Cell Culture, Portland, OR; 1:1) containing 1.05 mM calcium supplemented with 5 µg/ml crystalline bovine insulin (Sigma Aldrich, St. Louis, MO), 30 ng/ml cholera-toxin (Calbiochem, San Diego, CA), 2 ng/ml epidermal growth factor (EGF, R & D Systems, Inc., Minneapolis, MN), 0.5% dimethyl sulfoxide (DMSO, Sigma Aldrich), 0.5 µg/ml hydrocortisone, 5 ng/ml sodium selenite, and 5 µg/ml apo-transferrin, and supplemented with 10% fetal bovine serum (FBS). All reagents were obtained from Invitrogen Corporation (Grand Island, NY), except those indicated in the text [15,21]. B) The second was keratinocyte serum-free medium (KSFM) containing 0.09 mM calcium supplemented with 30 mg/ml pituitary bovine extract, 0.2 ng/ml EGF, 10% FBS, and ampicillin/streptomycin [22,23]. C) The third was Epilife medium (Cascade Biologics, Portland, OR), containing 0.06 mM calcium supplemented with 1% “human corneal growth supplement” (Cascade Biologics), containing 0.2% pituitary bovine extract, 5 g/ml bovine insulin, 0.18 mg/ml hydrocortisone, 5 μg/ml bovine transferrin, 0.2 ng/ml EGF, added 1% penicillin G sodium (Penicillin G sodium 10,000 g/ml, streptomycin sulfate 25 mg/ml, amphotericin B in 0.85% NaCl), and 5% FBS [8,24,25].

### Epithelial growth area and morphology

Cell morphology and the growth area of the limbal epithelial cells were determined in the three culture media conditions (ten different donor explants expanded in each of the three culture media). During the medium exchange, the margin of the epithelium adhered to the bottom of each culture plate was externally marked with a porous pen tip, delimiting the growth cell area. Subsequently, a transparent graph paper was placed under the sheet of the plate to count the previously defined area in mm^2^ [9].

### Immunocytochemistry

After 28 days of culture (five different donor explants expanded in each of the three culture media), the epithelial cells were washed three times with PBS (combination of sodium chloride, sodium phosphate, potassium chloride, potassium phosphate) and incubated with Trypsin/EDTA (EDTA) 0.5% (Invitrogen, Gibco Cell Culture, Portland, OR and Sigma Aldrich) at 37 °C for 5 min, and the detached cells were washed twice with the tested medium containing FBS [11]. Then the cells were placed in compartments of a Cytospin (Shandon, Pittsburgh, PA) and centrifuged at 900 rpm for 10 min to prepare slides containing 20,000 cells each. After centrifugation, the cells were dried for 15 min at room temperature, fixed in cold acetone for 1 min, and incubated for 10 min with blocking solution.

After being washed five times with PBS, the slides were incubated for 2 h in a moist chamber with the following primary monoclonal antibodies: Ki67 (1:100, Dako A/S, Glostrup, CPH) for cell proliferation [9,18]; cytokeratin 3 (CK3, 1:300, Chemicon International, Temecula, CA) for corneal epithelium [5,18,26]; adenosine 5′-triphosphate-binding cassette sub-family G member 2 (ABCG2, 1:50, R&D Systems, Emeryville, CA) for epithelial stem cells and progenitor cells [5,26]; p63 (1:50, Chemicon International), a putative epithelial stem-cell marker [5,18,26]; and vimentin (VMT, 1:300, R&D Systems) for mesenchymal cells [18]. Negative controls containing only antibody diluents were incubated in the same way.

After 2 h, the slides were washed with PBS, placed in a buffer bath for 5 min, and then incubated with amplification solutions of labeled streptavidin-biotin for 30 min each. The reactions were revealed with diaminobenzidine chromogen solution. The slides were then washed with distilled water, counter-stained with hematoxylin, and dehydrated with 100% alcohol for 15 min each, followed by a bath with xylol for 15 min. Slides were mounted with Entellan (Merck, Darmstad, HE) and coverslipped. The proportion of positive cells in each slide was determined by analyzing 200 cells/slide, using a light microscope (40x; Nikon Inc., New York, NY). All immunocytochemical reagents were obtained from Dako North America, Inc. (Carpinteria, CA) [5].

### Real-time reverse transcription polymerase chain reaction

To compare the gene expression of CK3, ABCG2, and p63 among the epithelial cells grown in the culture media, mRNA was extracted and analyzed with real-time PCR techniques after 28 days of culture (ten different donor explants expanded in each of the three culture media). Total RNA was obtained using the TRIzol kit (Invitrogen, Los Angeles, CA), which lyses cells and dissolves cell components, maintaining RNA integrity. The RNA quality was evaluated with electrophoresis in an agarose gel containing 1% formaldehyde for denaturing conditions.

The cDNA was obtained from the total RNA using the reverse transcriptase enzyme. The samples were treated with 1 U/μl of DNase. The reaction was performed in buffer solution with the specific enzyme for the reverse transcriptase enzyme, 0.5 mg/ml polythymine, 0.1 M of dithiothreitol, and 10 mM of each nucleotide. This solution was transferred to a tube containing 2 μg RNA, and finally, 1 μl of the reverse transcriptase enzyme was added. The reaction was performed at 42 °C for 50 min and stopped by raising the temperature to 70 °C for 15 min. The cDNA obtained was then used in PCR to amplify specific fragments of the CK3, p63, and ABCG2 gene.

The level of mRNA expression was estimated with quantitative real-time PCR using the GeneAmp 5700 Sequence Detection System SDS (ABI Prism 7700, Applied Biosystems, Branchburg, NJ), which was developed to detect gene expression with high specificity and sensitivity. The product obtained with real-time PCR was monitored using TaqMan (TaqMan Universal PCR Master Mix, Applied Biosystems, Branchburg, NJ). Relative expression of the gene was calculated using the logarithmic phase of amplification and was correlated to the number of initial copies of gene transcription. Fluorescence for each cycle was quantitatively analyzed with the ABI Prism 7700 SDS (Applied Biosystems). The relative amount of mRNA was estimated using a benchmark calculated from the control group as the average number of cycles determined by the threshold of all samples. The results for the experiments for each group were corrected by the expression of glyceraldehyde-3-phosphate dehydrogenase, which was used as the quantitative endogenous control and expressed in arbitrary units [5]. All PCR reagents were obtained from Invitrogen, except those indicated in the text.

### Hoechst staining

To evaluate and compare the devitalized throughout cells between the culture media, a quantitative method using Hoechst 33,342 dye (Sigma Aldrich) was applied. The cells were washed twice with PBS, trypsinized, and then neutralized with supplemented medium. The cells were collected in 1.5-ml tubes and centrifuged for 5 min at 1800 rpm, and the supernatant was discarded. An aliquot of 7 μl of the pellet was mixed in 3 μl of Hoechst solution, 10 mg/mL, for 10 min, placed on a slide and coverslipped. The slides were examined with a fluorescence microscope, and positive cells were expressed as a percentage [26].

### Statistical analysis

The Student *t* test and one-way ANOVA (ANOVA; SPSS ver.12, SPSS Inc. Chicago, IL), where non-parametric measure of correlation was performed using the Kruskal–Wallis test with Müller-Dunn’s post-test, were used to compare variables among the three groups. Probabilities of less than 5% were considered statistically significant. For repeated tests, we used the Bonferroni correction to correct for cumulative type I errors.

## Results

### Epithelial growth area and morphology

**Figure 1:**
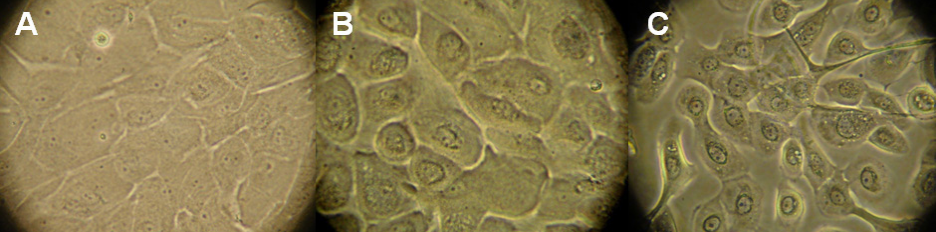
Optical morphological analysis of the limbal epithelial cells cultivated in three different culture media. **A**: supplemental hormonal epithelial medium (SHEM), **B**: keratinocyte serum-free medium (KSFM), and **C**: Epilife. Light microscope=40×.

**Figure 2:**
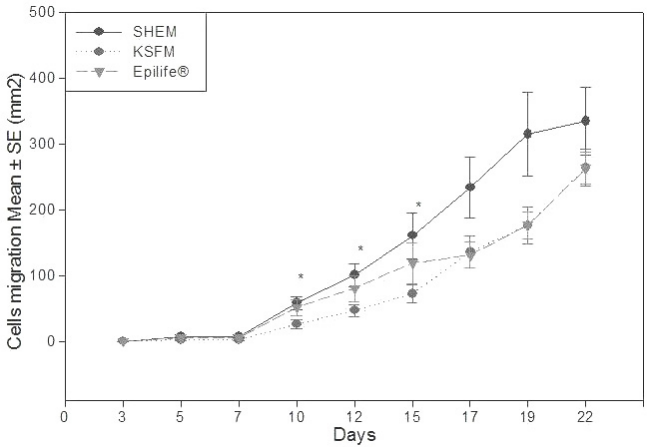
Plot of cell growth area over time for limbal epithelia cultivated in the culture media supplemental hormonal epithelial medium (SHEM), keratinocyte serum-free medium (KSFM), and Epilife. There were significant differences in cell growth area in SHEM and KSFM media at 10, 12, and 15 days of culture. One-way ANOVA was performed. Bars represent the standard deviation.

Optical morphological analysis of the limbal epithelial cells cultivated in three culture media is presented in Figure 1. The evolution of the cell growth area in the culture media is shown in Figure 2. In SHEM, the cells started to grow by 3–5 days, showed faster and progressive migration in 17–19 days of culture, and reached confluence in 24.61±8.35 days. The cells cultivated in this medium were initially smaller and better defined, with a 1:2 nucleus-cytoplasm ratio. As migration occurred, the cell layer demarcation became clear with a 1:3, 1:4 nucleus-cytoplasm ratio.

In KSFM, the cells started to grow in 3–5 days, showed faster migration at 19–22 days of culture, and reached confluence in 27.28±4.41 days. The cells grown in this medium displayed epithelial characteristics, a 1:2 nucleus-cytoplasm ratio, but as soon as the migration started, they started to show a 1:3 nucleus-cytoplasm ratio.

The cells cultured in Epilife medium grew more slowly; growth began after 3–10 days. Cell growth reached a peak after 19–22 days of culture, and total confluence occurred after 29.22±5.35 days. The cells grown in this medium were more uniform, with a better definition of cell boundaries and a cuboidal appearance with a 1:2 nuclear-cytoplasm ratio.

The cell growth area comparison can be better observed in Figure 2 and Table 1. Significant differentiation in the growth area rate can be seen on days 10, 12, and 15 of cell culture.

**Table 1:** Cell migration of limbal epithelia cultivated in the culture media SHEM, KSFM and Epilife®

Days in culture	SHEM Mean	KSFM Mean	Epilife	P value
3	0.13±0.35	0.13±0.35	0	0.5929
5	7.89±2.67	2.67±4.42	5.44±3.07	0.1146
7	7.89±8.66	2.67±4.42	5.44±3.07	0.1146
10	58.40±30.20	26.10±18.70	51.40±37.00	0.0317*
12	100.80±52.90	46.90±27.80	80.40±58.60	0.0275*
15	160.50±104.30	72.20±39.5	119.00±92.80	0.0440*
17	233.40±139.50	136.10±71.75	130.90±59.64	0.1711
19	314.80±190.50	176.20±82.20	176.40±61.50	0.291
22	334.10±144.80	263.90±84.30	262.70±71.80	0.6388

SD=standart deviation; SHEM=supplemental hormonal epithelial medium; KSFM=keratinocyte serum-free medium; One way ANOVA was performed.

### Immunocytochemistry

**Figure 3:**
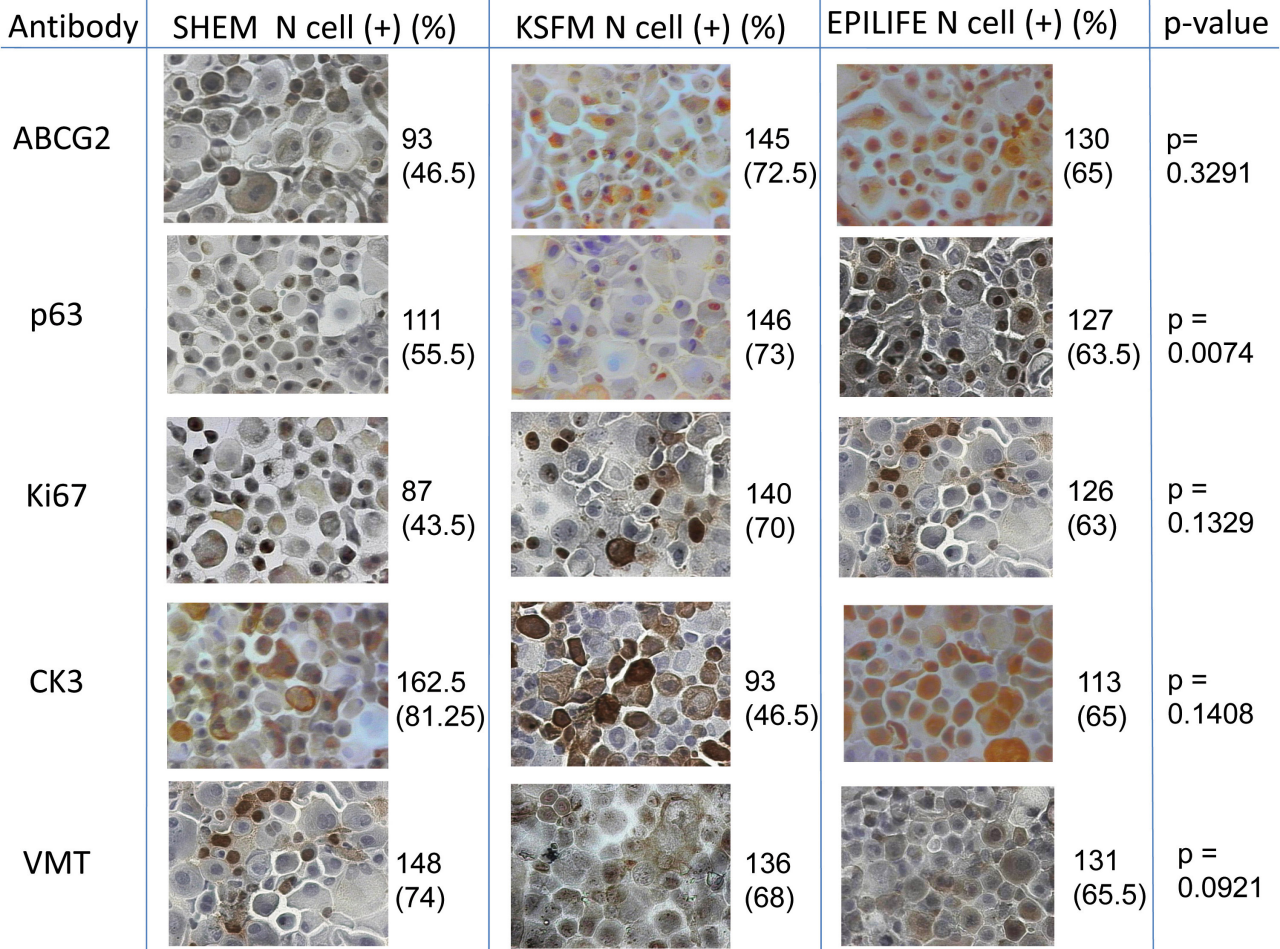
Immunocytochemistry of limbal epithelial cells cultured in supplemental hormonal epithelial medium (SHEM), keratinocyte serum-free medium (KSFM) and Epilife showing the number of positive cells (N cell +) and percentage of positive cells. The Kruskal–Wallis test was performed. There were significant differences for p63. (Light microscope 40x).

Immunocytochemical analysis (Figure 3) showed that the cells grown in SHEM had a lower percentage of positive cells for putative stem cell markers (ABCG2 and p63) compared to KSFM and Epilife. As for cell proliferation, SHEM showed a lower percentage of Ki67 compared to KSFM and Epilife. For differentiated epithelium markers, a higher percentage of CK3 was found with SHEM; however, KSFM and Epilife had a lower tendency for CK3-positive cells. SHEM-cultivated cells also showed a higher propensity of positive cells for VMT compared to the other groups.

### Real-time reverse transcription polymerase chain reaction

**Figure 4:**
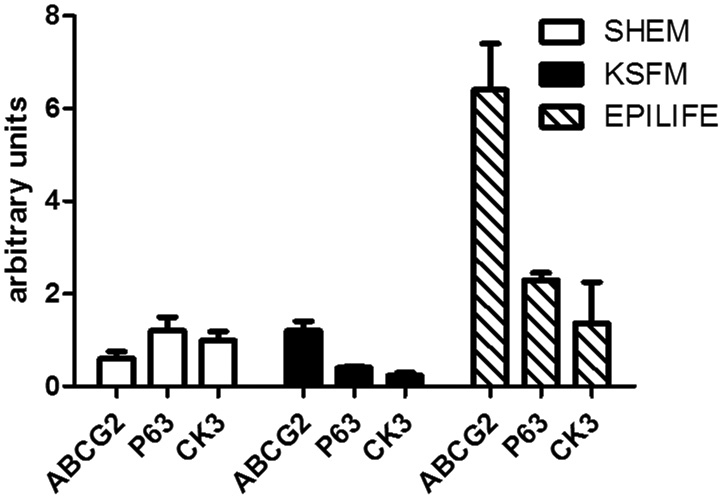
Expression level of cell differentiation marker CK3, stem cell marker ABCG2 and undifferentiated cell marker p63, determined with reverse transcription polymerase chain reaction (RT–PCR) of limbal epithelial cells cultured in supplemental hormonal epithelial medium (SHEM), keratinocyte serum-free medium (KSFM), and Epilife. One way ANOVA was performed.

The results of real-time PCR analysis are presented in Figure 4. ABCG2 and p63 expression was statistically higher for Epilife (p=0.001, one-way ANOVA) compared to SHEM and KSFM. However, CK3 expression was statistically lower for KSFM (p=0.039, one-way ANOVA) compared to SHEM and Epilife.

### Cell viability

Assays for cells devitalized throughout with Hoechst 33,342 showed that cells in the isolated corneal–limbal epithelial sheets remained viable. There was no significant difference in the proportion of devitalized throughout cells among the three culture media (one-way ANOVA, p=0.9253) presented in Table 2.

**Table 2:** Percentage of devitalized throughout cells with Hoechst staining

Days in culture	SHEM Mean	KSFM Mean	Epilife	P value
Hoechst	60	75	51	0.9253
	−30	−37.5	−25.5	

N cells +=number of positive cells; SHEM=supplemental hormonal epithelial medium; KSFM=keratinocyte serum-free medium; Kruskal–Wallis test was performed.

## Discussion

The present study provides evidence that limbal epithelial cells grew differently in the three tested culture media and showed different phenotypes and expression of markers for putative progenitor cells and differentiated cells. In SHEM, growth and confluence occurred earlier, around 24 days of culture when compared to the other groups. As confluence progressed, the cell size and nuclear size increased. Cell proliferation was significantly higher at 12–19 days, but decreased at the end of the experiment to levels no longer significant. This decrease in the proliferation rate was confirmed with the lower percentage of positive cells for Ki67 (43.5%), a cell proliferation marker, in immunocytochemistry. These findings suggest that cultured cell were in the process of higher differentiation.

KSFM and Epilife showed significantly slower growth compared to SHEM between 17 and 22 days. Nevertheless, Ki67 gene expression was high at the 28th day of culture. The smaller cell size and slower proliferation observed in KSFM and Epilife are compatible with the less differentiated epithelium reported by Romano et al. [27], who used a combination of in vivo confocal microscopy and flow cytometry to characterize the limbal basal epithelium.

ABCG2, a member of the ABC transporters, formally known as breast cancer resistance protein 1 (BCRP1), has been identified as a molecular determinant for bone marrow SCs, and has been proposed as a universal SC marker [28]. Similarly, the nuclear transcription factor p63, a member of the p53 family, was proposed as a marker of corneal epithelial SCs. In all tested culture media, gene expression for putative progenitor cells markers was detected with RT–PCR and immunocytochemistry. Although ABCG2 and p63 protein expression did not present discrepant differences among the three culture media conditions tested, our findings demonstrate that Epilife better maintained the progenitor cell characteristic due to higher mRNA expression of ABCG2 and p63 when compared to KSFM and SHEM at the 28th day of culture. The immunocytochemistry study also presented higher protein expression for ABCG2 and p63 for the Epilife culture. Immunocytochemistry and RT–PCR studies were also comparable for lower signal from cells in SHEM. In KSFM, however, higher expression on immunocytochemistry was dissociated from low mRNA detection for progenitor cell markers. This may represent protein synthesis in earlier stage of cell culture with less mRNA activity at the 28th day of culture. In contrast, all groups, excepting KSFM with a lower signal, expressed CK3, indicating the presence of differentiated cells. Although Epilife seemed to be more effective in maintaining the limbal epithelial progenitor cells phenotype, the cultures were heterogeneous, and we found CK3-positive cells.

However, all groups expressed CK3, indicating the presence of differentiated cells, and the KSFM was the medium that less expressed CK3. Although KSFM and Epilife seemed to be more effective in maintaining the limbal epithelial progenitor cells phenotype, the cultures were heterogeneous, and we found CK3-positive cells.

In this study, we did not evaluate the colony-forming efficiency in culture media described by Pellegrini [13] in cultures established with the cell suspension technique. Nevertheless, we followed the concept described by Li et al. and others [26,29] that proposes that human limbal epithelial cells with stem cell properties express high levels of ABCG2 RNA, and this fact correlates with the greatest colony-forming efficiency and growth capacity.

A notable difference among culture media components is the calcium concentration. KSFM and Epilife, which kept cells in a more undifferentiated state, presented a lower calcium concentration than SHEM.

The influence of calcium in cellular differentiation has been known for 30 years and was reported by Boyce and Ham [30] and other authors [22,31–33]. These authors have shown that the presence of a high extracellular calcium concentration produces a stimulatory effect on cell proliferation, because the calcium interacts with the mitogenic effects of growth factors, such as EGF. Moreover, Kruse et al. [33] reported that calcium can also induce keratinocytes to synthesize transforming growth factor-β (TGF-β) mRNA promoting epithelial differentiation. Nevertheless, the presence of TGF-β produces a negative effect on cell proliferation induced by EGF. In this study, at the initial stage of the culture, the proliferation of limbal epithelial cells probably occurred due to the interaction with EGF and the high calcium concentration, and in a more advanced stage of culture, the cells start to produce TGF-β, causing a decline in cell proliferation. The release of TGF-β may also occur in prolonged culture life or high cell density [33]. In addition, some authors described that only in cultures with a high calcium concentration abnormal differentiation with expression of cornified envelopes can be found [22,31–34].

Transplantation of cultured epithelium that presents more differentiated cells may perceive short-term success due to better cellular adhesion by desmosomes. However, it has the disadvantage of reducing the stemness of the transplanted cells, which can compromise the viability of new epithelia formed in the long term. Sangwan and colleagues [4] demonstrated the clinical benefit of using monolayer limbal epithelial cells transplanted in a lower differentiation condition, since the authors were able to induce a healthy and longstanding stratified epithelium.

Others authors have shown that epithelial cells cultured in low calcium concentration media had a cobblestone-like morphology in a monolayer, with well defined edges, reflecting a lack of calcium needed in the desmosome formation for cell adhesion [22,31–33]. In the present study, we observed that limbal epithelial progenitor cells cultured in KSFM and Epilife began to proliferate, even with low calcium. These results should be addressed in future studies.

Another important component of culture media is FBS, which stimulates epithelial growth [22,35]. The influence of FBS concentration on epithelial growth was described by Kruse et al. [33], who demonstrated that adding FBS at a high concentration (10%–20%) stimulates the proliferation of limbal epithelial cells. The calcium presented in the FBS has significant influence on the final calcium concentration in the culture media. Furthermore, the TGF-β and other components present in the FBS can stimulate cell differentiation. Nevertheless, the differences in the calcium concentrations among the media used in the present study were maintained with a higher concentration for SHEM, followed by KSFM, and Epilife with the lowest concentration of calcium, as measured preliminarily.

Although some of the immunocytochemistry and PCR results were not statistically significant and despite the different trend profiles of cell differentiation in the culture media tested, they all exhibited progenitor cells even after 30 days of culture. Clinical studies comparing epithelial cells in different culture media with various stem cell profiles should still be tested.

The use of a 3T3 feeder layer is a precondition for cultivating and expanding limbal epithelial progenitor cells using the cell suspension technique. Varghese et al. [36] showed that in culture with the limbal explant without 3T3 feeder layer fibroblast-like cells form, which probably migrate out from the limbal stroma and may act as a feeder layer for limbal epithelial progenitor cells. Ghoubay-Benallaoua et al. and Ma et al. [37,38] described that the cholera toxin used in SHEM is capable of inhibiting the growth of fibroblasts in culture. However, in the current study, we observed the presence of fibroblast-like cells and expression of VMT, a marker of mesenchymal cells, in all groups, even in SHEM, suggesting that fibroblasts were growing in the cultures. The presence of fibroblast may mimic natural tissue architecture and niche by enabling the formation of an auto-feeder layer and thereby eliminating the need for xeno-feeder layers for ex vivo expansion of epithelial progenitor cells. This may represent a safety advantage compared to the cell suspension techniques proposed by Pellegrini and others [11,36], since the xenogenic component in the culture may present a risk of cross-transfer of pathogens that limits medical application. Future research should address the influence of culture media in stem cell proliferation cocultured with autogenic fibroblast cells.

In summary, cells cultured in KSFM and Epilife presented a higher percentage of limbal epithelial progenitor cells when compared to SHEM. More studies are needed to further characterize the ideal culture condition for limbal epithelial progenitor cells to be used for ex vivo transplantation in ocular surface reconstruction.
